# ﻿Multi-gene phylogenetic analyses revealed two novel species and one new record of *Trichobotrys* (Pleosporales, Dictyosporiaceae) from China

**DOI:** 10.3897/mycokeys.106.123279

**Published:** 2024-06-20

**Authors:** Wen-Jing Zhang, Gui-Ping Xu, Yu Liu, Yang Gao, Hai-Yan Song, Hai-Jing Hu, Jian-Ping Zhou, Ming-Hui Chen, Deng-Mei Fan, Dian-Ming Hu, Zhi-Jun Zhai

**Affiliations:** 1 College of Bioscience and Bioengineering, Jiangxi Agricultural University, Nanchang, 1101 Zhimin Road, Nanchang, 330045, China; 2 School of Biology and Biological Engineering, South China University of Technology, Guangzhou, 510006, China; 3 Key Laboratory of Industrial Ecology and Environmental Engineering (Ministry of Education), School of Ocean Science and Technology, Dalian University of Technology, Panjin Campus, China; 4 Jiangxi Key Laboratory for Excavation and Utilization of Agricultural Microorganisms, Jiangxi Agricultural University, Nanchang, 1101 Zhimin Road, Nanchang, 330045, China; 5 School of Agricultural Sciences, Jiangxi Agricultural University, Nanchang, 1101 Zhimin Road, Nanchang, 330045, China

**Keywords:** Freshwater hyphomycetes, phylogenetic analysis, taxonomy, *Trichobotrys*

## Abstract

The rotting wood in freshwater is a unique eco-environment favoring various fungi. During our investigation of freshwater fungi on decaying wood, three hyphomycetes were collected from Jiangxi and Guangxi Provinces, China. Based on the morphological observations and phylogenetic analysis of a combined DNA data containing ITS, LSU, SSU and *tef1-α* sequences, two new *Trichobotrys* species, *T.meilingensis* and *T.yunjushanensis*, as well as a new record of *T.effusa*, were introduced. Additionally, a comprehensive description of the genus with both morphological and molecular data was first provided.

## ﻿Introduction

*Trichobotrys* Penzig & Saccardo is a genus introduced with the discovery of the type species *Trichobotryseffusa* (Berk. & Br.) Petch from Sri Lanka, which was placed in Pleosporales genera *incertae sedis* (Pleosporales, Dothideomycetes, Ascomycota) (Petch 1924; Morgan-Jones et al. 1987). *Trichobotryseffusa* is known for producing compounds which can exhibit significant growth-inhibitory activities against the A549 lung cancer cell line (Chen et al. 2014). In addition, the bioactive compounds obtained from the deep-sea-derived fungus *T.effusa* DEFSCS021 could strongly inhibit the larvae settlement of *Bugulaneritina* and *Balanusamphitrite* larvae (Sun et al. 2016).

*Trichobotrys* encompasses fungi characterised by their mononematous conidiophores producing catenate, dark brown, spherical and echinulate conidia on fertile, smooth, short, lateral branches with polyblastic conidiogenous cells. So far, only five species are recognised in this genus (http://www.indexfungorum.org/Names/Names.asp), namely *T.effusa*, *T.ipomoeae*, *T.pannosa*, *T.ramosa* and *T.trechispora*. However, *T.pannosa* has been treated as a synonym of *T.effusa* (Morgan-Jones et al. 1987; D’Souza and Bhat 2001). Therefore, *Trichobotrys* is supposed to comprise four saprobic species, of which one (*T.effusa*) is from aquatic habitats and three (*T.ipomoeae*, *T.ramosa* and *T.trechispora*) are from terrestrial habitats (Petch 1917, 1924; Sawada 1959; Morgan-Jones et al. 1987; D’Souza and Bhat 2001). To date, the phylogenetic positions of representatives of *Trichobotrys* within the Ascomycota have not yet been investigated, as *T.effusa* has only ITS sequence and there are no molecular data for *T.ipomoeae*, *T.ramosa* and *T.trechispora*.

In the current study, we attempt to clarify the classification status of *Trichobotrys* through further identified materials and a more appropriate multi-gene genealogy. During our investigation of the freshwater hyphomycetes from decaying wood in Jiangxi and Guangxi provinces of China, two novel species named *T.meilingensis* and *T.yunjushanensis*, as well as a new record of *T.effusa*, are described according to morphological examination and multi-loci phylogenetic evidence.

## ﻿Materials and methods

### ﻿Samples collection, morphological observation and isolation

Samples of dead wood submerged in freshwater streams were collected from Jiangxi and Guangxi Provinces, China and were brought to the laboratory in plastic bags. Observations for fungi on natural substrates were made using a Nikon SMZ-1270 microscope (Nikon Corporation, Japan). With a syringe needle, the fungal structures were gathered and transferred to a small drop of distilled water on a clean slide, which was covered with a cover slide (Yang et al. 2018a). Micro-morphological characters were observed by a Nikon ECLIPSE Ni-U compound microscope (Nikon Corporation, Japan) and photographed by a Nikon DS-Fi3 camera. All measurements of the fungal structures were performed with PhotoRuler v. 1.1 software (The Genus Inocybe, Hyogo, Japan) and figures were made with Adobe Photoshop CC 2017 software (Adobe Systems, USA). Pure cultures of the fungi were obtained by the single spore isolation method (Chomnunti et al. 2014). Germinating conidia were transferred to fresh potato dextrose agar (PDA, from Beijing Bridge Technology Co., Ltd., Beijing, China) supplemented with two types of antibiotics (100 μg/mL penicillin, 50 μg/mL streptomycin), and then incubated at 25 °C for 2–3 weeks. Pure cultures were deposited at the Jiangxi Agricultural University Culture Collection (JAUCC) and specimens were stored in the Herbarium of Fungi, Jiangxi Agricultural University (HFJAU).

### ﻿DNA extraction, PCR amplification and sequencing

Fresh mycelia of each strain, scraped from the growing culture with a sterile scalpel, were ground to a fine powder with liquid nitrogen to break the cells for DNA extraction. Subsequently, total genomic DNA was extracted following the modified CTAB method (Doyle and Doyle (1987). Four primer pairs, ITS1/ITS4 (White et al. 1990), LR0R/LR7 (Hopple and Vilgalys 1999), NS1/NS4 (White et al. 1990) and EF1-983F/EF1-2218R (Rehner and Buckley 2005), were used to amplify ITS, LSU, SSU and *tef1-α* gene regions, respectively. Polymerase chain reaction (PCR) was performed in a final volume of 25 μl, containing 9.5 μl double distilled water (ddH_2_O), 12.5 μl 2 × Taq PCR MasterMix (Qingke, Changsha, China), 1 μl each primer (10 μM) and 1 μl genomic DNA extract. Amplification conditions for ITS, LSU, SSU and *tef1-α* gene regions followed Zhai et al. (2022). The PCR products were sent to be sequenced by the commercial company QingKe Biotechnology Co. (Changsha, China). All sequences were edited with SeqMan v. 7.1.0 (DNASTAR, lnc, Madison, WI) and were deposited in the NCBI GenBank database (Table 1).

**Table 1.** Sequences used in this study.

Species	Isolate	GenBank accession number			
		ITS	LSU	SSU	*tef1-α*
***Aquadictyosporaclematidis***	**MFLU 172080**	**MT310592**	**MT214545**	**MT226664**	**MT394727**
*Aquadictyosporalignicola*	MFLUCC 17-1318	MF948621	MF948629	–	MF953164
***Dendryphiellaparavinosa***	**CPC 26176**	**KX228257**	**KX228309**	–	–
*Dendryphiellavinosa*	MFLU 200444	MT907477	MT907480	–	–
***Dictyocheirosporaaquatica***	**KUMCC 15-0305**	**KY320508**	**KY320513**	–	–
***Dictyocheirosporabannica***	**KH 332**	**LC014543**	**AB807513**	**AB787223**	**AB808489**
*Dictyocheirosporabannica*	MFLU 18-1040	MH381765	MH381774	MH381759	–
***Dictyocheirosporagarethjonesii***	**MFLUCC 16-0909**	**KY320509**	**KY320514**	–	–
*Dictyocheirosporagarethjonesii*	DLUCC 0848	MF948623	MF948631	–	MF953166
*Dictyocheirosporapseudomusae*	yone 234	LC014550	AB807520	AB797230	AB808496
*Dictyocheirosporapseudomusae*	KH 412	LC014549	AB807516	AB797226	AB808492
*Dictyocheirosporaheptaspora*	DLUCC 1992	MT756244	MT756243	–	MT776563
***Dictyocheirosporarotunda***	**MFLUCC 14-0293**	**KU179099**	**KU179100**	**KU179101**	–
*Dictyocheirosporarotunda*	MFLUCC 17-0222	MH381764	MH381773	MH381758	MH388818
***Dictyosporiumalatum***	**ATCC 34953**	**NR–077171**	**DQ018101**	**DQ018080**	–
*Dictyosporiumbulbosum*	yone 221	LC014544	AB807511	AB797221	AB808487
*Dictyosporiumdigitatum*	KT 2660	LC014546	AB807518	AB797228	–
*Dictyosporiumdigitatum*	KH 401	LC014545	AB807515	AB797225	AB808491
*Dictyosporiumdigitatum*	yone 280	LC014547	AB807512	AB797222	AB808488
*Dictyosporiumelegans*	NBRC 32502	DQ018087	DQ018100	DQ018079	–
*Dictyosporiumhughesii*	KT 1847	LC014548	AB807517	AB797227	AB808493
*Dictyosporiummeiosporum*	MFLUCC 10-0131	KP710944	KP710945	KP710946	–
*Dictyosporiumnigroapice*	MFLUCC 17-2053	MH381768	MH381777	MH381762	MH388821
***Dictyosporiumolivaceosporum***	**KH 375**	**LC014542**	**AB807514**	**AB797224**	**AB808490**
*Dictyosporiumpandanicola*	MFLUCC 18-0331	MZ490792	MZ490776	–	MZ501208
***Dictyosporiumstellatum***	**CCFC 241241**	**NR–154608**	**JF951177**	–	–
***Dictyosporiumstrelitziae***	**CBS 123359**	**NR–156216**	**FJ839653**	–	–
*Dictyosporiumtetrasporum*	KT 2865	LC014551	AB807519	AB797229	AB808495
*Dictyosporiumthailandicum*	MFLUCC 13-0773	KP716706	KP716707	–	–
***Dictyosporiumtratense***	**MFLUCC 17-2052**	**MH381767**	**MH381776**	**MH381761**	**MF388820**
*Digitodesmiumbambusicola*	CBS 110279	DQ018091	DQ018103	–	–
***Gregaritheciumcurvisporum***	**KT 922**	**AB809644**	**AB80754**	**AB797257**	**AB808523**
***Jalapriyapulchra***	**MFLU 17-1683**	**MF948628**	**MF948636**	–	**MF953171**
*Jalapriyatoruloides*	CBS 209.65	DQ018093	DQ018104	DQ018081	–
*Periconiaigniaria*	CBS 379.86	LC014585	AB807566	AB797276	AB808542
*Periconiaigniaria*	CBS 845.96	LC014586	AB807567	AB797277	AB808543
***Pseudocoleophomacalamagrostidis***	**KT 3284**	**LC014592**	**LC014609**	**LC014604**	**LC014614**
*Pseudocoleophomaflavescens*	CBS 178.93	–	GU238075	GU238216	–
***Pseudocoleophomapolygonicola***	**KT 731**	**AB809634**	**AB807546**	**AB797256**	**AB808522**
*Pseudocoleophomazingiberacearum*	NCYUCC 190054	MN615941	MN616755	–	MN629283
***Pseudodictyosporiumelegans***	**CBS 688.93**	**MH862454**	**MH874101**	**DQ018084**	–
***Pseudodictyosporiumthailandica***	**MFLUCC 16-0029**	**KX259520**	**KX259522**	**KX259524**	**KX259526**
***Pseudodictyosporiumwauense***	**CBS 126094**	**MH864014**	**MH875472**	–	–
*Trichobotryseffusa*	FS524	MN545626	–	–	–
*Trichobotryseffusa*a	YMJ1179	KJ630313	–	–	–
Trichobotryseffusa*	JAUCC 6359	PP406377	PP407503	PP407508	PP405621
Trichobotryseffusa*	JAUCC 6826	PP830649	PP830650	PP830652	PP845300
Trichobotrysmeilingensis*	**JAUCC 4985**	**PP406380**	**PP407504**	**PP407509**	**PP405623**
Trichobotrysmeilingensis*	JAUCC 4986	PP406381	PP407505	PP407510	PP405625
Trichobotrysyunjushanensis*	**JAUCC 4987**	**PP406378**	**PP407506**	**PP407511**	**PP405622**
Trichobotrysyunjushanensis*	JAUCC 4988	PP406379	PP407507	PP407512	PP405624

Ex-type strains or type materials are marked in bold. Newly generated sequences are indicated with “*”. “–”, the sequence is unavailable.

### ﻿Data analyses

Based on ITS, LSU, SSU and *tef1-α* sequence comparison with the GenBank database, similar species in Dictyosporiaceae were found. The sequences of 37 relevant species according to the blasting result and recent publications (Tanaka and Harada 2003; Chen et al. 2014; Tanaka et al. 2015; Boonmee et al. 2016; Liu et al. 2017; Yang et al. 2018a; Chen et al. 2020) were chosen for phylogenetic analyses (Table 1) and were downloaded from GenBank. Four gene regions (ITS, LSU, SSU and *tef1-α*) were individually aligned using the online service of MAFFT v. 7 (Madeira et al. 2019) and concatenated using PhyloSuite v. 1.2.2 (Zhang et al. 2020). The alignments were checked visually and improved manually using BioEdit (Hall 1999; Liu et al. 2017).

Maximum Likelihood (ML) and Bayesian Inference (BI) were used to assess phylogenetic relationships. Maximum Likelihood (ML) analysis was conducted with RAxML v. 7.2.6 (Stamatakis and Alachiotis 2010) using the default substitution model GTR-GAMMA with rapid bootstrap analysis followed by 1000 bootstrap replicates to estimate ML bootstrap (BS) values. Bayesian Inference (BI) analysis was carried out with MrBayes v. 3.2 under partitioned models (Ronquist et al. 2012). The best-fit models of nucleotide substitutions were selected according to the Akaike information criterion (AIC) implemented in jModelTest v. 2.1.1 (Darriba et al. 2012) on XSEDE in the CIPRES web portal (Miller et al. 2010). The models for ITS, LSU, SSU and *tef1-α* datasets used for phylogenetic analysis are TIM2+I+G model (-lnL = 5321.6598), TIM2+I+G model (-lnL = 3199.3778), TIM2+I+G model (-lnL = 3481.7971) and GTR+I+G model (-lnL = 4762.6993), respectively. The data sets were run for 10,000,000 generations, with four chains, sampling trees every 1,000 generations. The first 10% trees were discarded as burn-in. Phylogenetic trees were visualized with FigTree v. 1.4.4 (Rambaut 2018), edited and beautified using Adobe Illustrator 2020 (Adobe Systems Inc., USA).

## ﻿Results

### ﻿Molecular phylogenetic results

According to sequence alignment analysis, the ITS sequences of the new record *Trichobotryseffusa* (JAUCC 6359 and JAUCC 6826) have only two different loci from that of *T.effusa* FS524 and three loci from that of *T.effusa* YMJ1179. The aligned sequence matrix for the combined analysis consists of ITS (574 bp), LSU (1259 bp), SSU (1459 bp) and *tef1-α* (962 bp) with a total of 4254 characters including gaps. The combined dataset shows the new species *T.meilingensis* and *T.yunjushanensis* share 98.61% (59 different loci), 98.40% (68 different loci) sequence similarity with *T.effusa* (JAUCC 6359 and JAUCC6826), respectively, but are less similar to *Gregaritheciumcurvisporum* [95.75% (181 different loci) and 95.53% (190 different loci), respectively]. In addition, there are 57 different loci between the sequences of the two new species.

**Figure 1.**
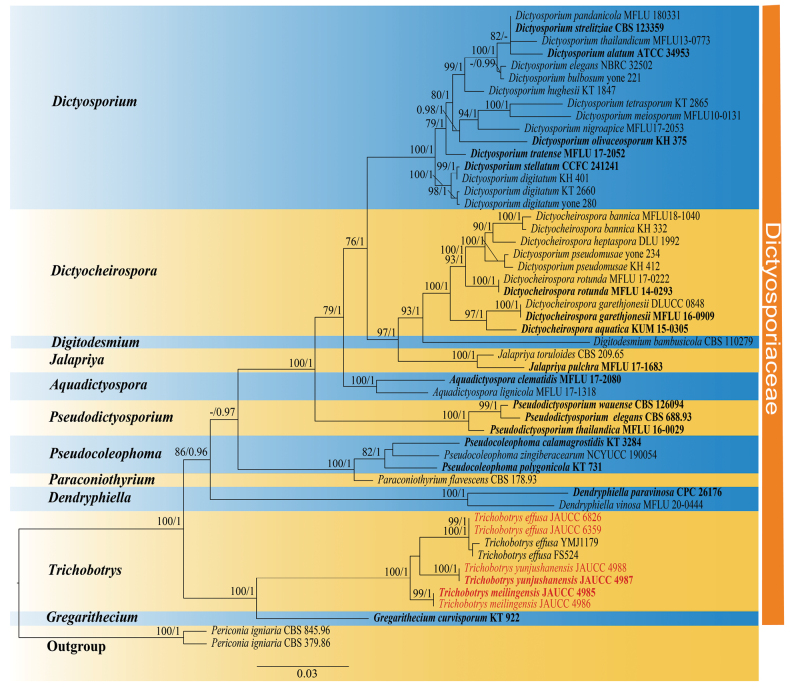
Phylogenetic tree of Dictyosporiaceae inferred from the combined regions (ITS-LSU-SSU-*tef1-α*) using Maximum Likelihood (ML) analysis. The *Periconiaigniaria* clade was used as the outgroup. PP ≥ 0.95 and BS ≥ 75% were indicated around the branches. The new sequences generated in this study are given in red and type strains are in bold.

The topologies of the phylogenetic trees produced by ML and BI are congruent, and the best RAxML tree with BS and PP is shown in Fig. 1. Phylogenetic analyses indicate that the new *Trichobotryseffusa* isolates (JAUCC 6359 and JAUCC 6826) cluster with other *T.effusa* collections (FS524 and YMJ1179) in a strongly-supported monophyletic clade (BS/PP = 100/1). Moreover, *T.yunjushanensis* is sister to the *T.effusa* clade, but only with low ML bootstrap support values (BS = 43) and Bayesian posterior probabilities (PP = 0.67). However, these two species and *T.meilingensis* form a well- supported clade (BS/PP = 100/1), which is phylogenetically close to *Gregaritheciumcurvisporum* (BS/PP = 100/1).

### ﻿Taxonomy

#### ﻿ Trichobotrys meilingensis

Taxon classificationFungiPleosporalesDictyosporiaceae

G. P. Xu & Z. J. Zhai sp. nov.

63081B33-2479-53BE-B217-3BC1E4B3DF6F

852617

Fig. 2

##### Etymology.

Referring to the collection site of the Meiling Mountain in Jiangxi Province, China.

##### Holotype.

HFJAU10042.

##### Description.

Saprobic on the stems of bamboo in freshwater habitats. **Sexual morph**: Undetermined. **Asexual morph**: Hyphomycetous. ***Colonies*** effuse, white to yellow, hairy. ***Mycelium*** partly superficial, partly immersed, gregarious and creeping, composed of septate, branched, pale brown hyphae. ***Conidiophores*** 2.5–4.5 μm wide (x̄ = 3.5 μm, n = 20), up to 510 μm long, mononematous, variously curved, dichotomously branched in the conidiophore, septate, thick-walled, verruculose, echinulate, brown to dark brown. ***Conidiophore branches*** 15–39 × 3–4 μm (x̄ = 24.5 × 3.4 μm, n = 15), fertile, 0‒1(‒2)-septate, verruculose, pale to dark brown. ***Conidiogenous cells*** 7–12 × 3–5 μm (x̄= 9.0 × 4.0 μm, n = 10), polyblastic, integrated, erect or curved, widely distributed in the fertile branches, denticulate, hyaline to brown. ***Conidia*** 7‒13 μm diam (x̄ = 9.8 μm, n = 30), catenate, usually in branched, acropetal chains, aseptate, globose, verruculose, echinulate, sometimes guttulate, yellow brown to dark brown.

**Figure 2.**
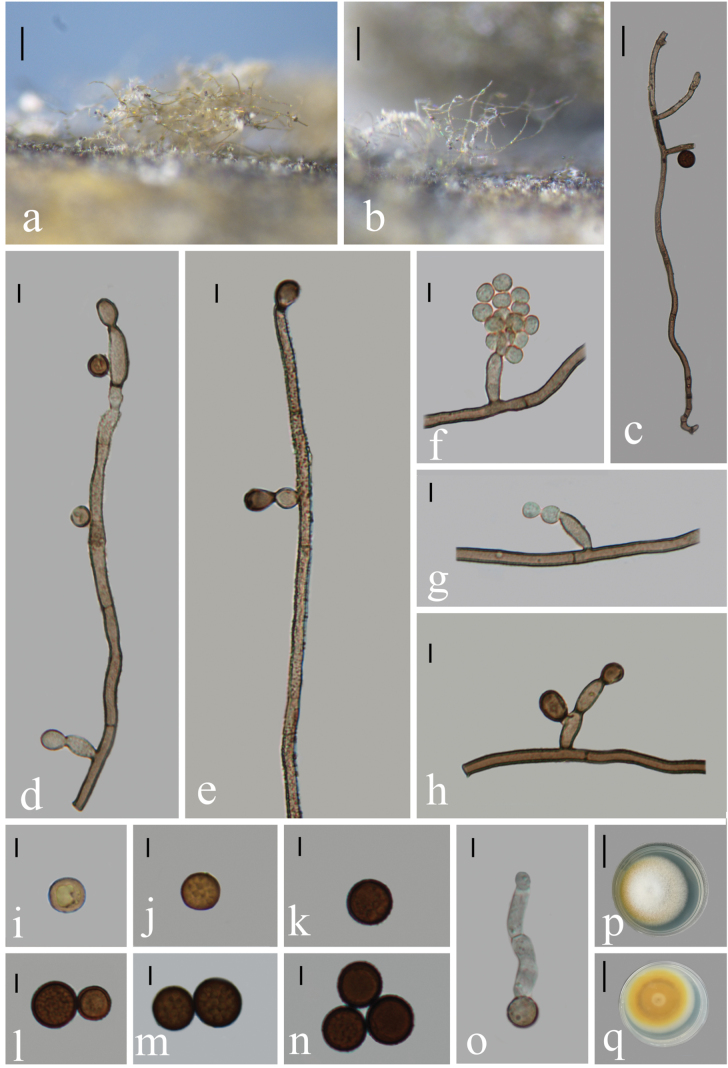
*Trichobotrysmeilingensis* (HFJAU10042, holotype) **a, b** colonies on bamboo culms **c–e** conidiophores with conidiogenous cells **f** portion of conidiophore with fertile lateral branches **g, h** conidiogenous cells **i–n** conidia **o** germinating conidium **p, q** culture on PDA from above (**p**) and reverse (**q**). Scale bars: 100 µm (**a, b**); 20 µm (**c**); 5 µm (**d‒o**); 25 mm (**p, q**).

##### Cultural characteristics.

Conidia germinating on PDA within 24 h. Colonies incubated on PDA media at 25 °C attaining 30.5 mm diam after 9 days, in natural light, circular, white, slightly cottony, yellow at the margin part, with white dense aerial mycelium; reverse yellow, white at the entire margin.

##### Material examined.

China. Jiangxi Province: Nanchang City, Meiling Mountain, on decaying bamboo culms submerged in a freshwater stream, alt. 305 m, near 28.79°N, 115.72°E, 16 August 2021, G. P. Xu, Y. Liu and Z. J. Zhai, SLT-32 (HFJAU10042, ***holotype***), ex-type living culture, JAUCC 4985 = JAUCC 4986.

##### Notes.

*Trichobotrysmeilingensis* is similar to other species of *Trichobotrys* in having monomatous conidiophores, spherical and echinulate conidia, and polyblastic conidiogenous cells. *Trichobotrysmeilingensis* is easily distinguished from *T.effusa*, *T.ipomoeae* and *T.trechispora* by its dichotomously branched conidiophores and its conidial size (7‒13 μm vs. 3‒4 μm, 13‒15.5 μm and 3‒5 μm, respectively) (Petch 1917, 1924; Sawada 1959). *Trichobotrysmeilingensis* is morphologically most similar to *T.ramosa* and shares some characteristics, such as dichotomously branched conidiophores and catenate conidia. However, *T.meilingensis* has larger conidia (7–13 μm vs. 3‒5 μm) and thinner conidiophores (2.5‒4.5 μm vs. 8‒18 μm) (D’Souza and Bhat 2001). Therefore, *T.meilingensis* can be distinguished from *T.ramosa* based on morphological characters in spite of the unavailable molecular data of the latter species. Thus, it should be identified as an independent taxon in *Trichobotrys*. A comparison of morphological features of *Trichobotrys* species is provided in Table 2.

**Table 2.** Synopsis of morphological characteristics, habitats, hosts and district compared across *Trichobotrys* species.

Species	Conidiophores (μm)	Conidia (μm)	Conidiophores characteristics	Conidia characteristics	Habitat	Host	District	References
*Trichobotryseffusa*	Up to 200 × 3–4 or up to 1000 × 4–6	3–4 or 5–7	Equal, septate, with short lateral branches, thick walled, minutely verrucose	Globose, red-brown or brown, minutely verrucose	Freshwater	On fallen leaves of dead bamboo or decorticated wood	Sri Lanka and South Africa	Berkeley and Broome (1873); Petch (1924); Morgan-Jones et al. (1987)
***T.effusa***	**Up to 650 × 2–4**	**3.5–5**	**Mononematous, erect, with short lateral branches, verruculose, septate, thick-walled, light brown to nut brown**	**Spherical, verruculose, echinulate, transparent to dark brown or red brown**	**Freshwater**	**On Dead wood**	**China, Guangxi**	**This study**
*T.ipomoeae*	195–440 × 13–16	13–15.5	Simple, cylindrical, 2–3 septate, dark brown	Spherical, verrucose, brown	Terrestrial	On the leaves of *ipomoea pescaprae*	China, Taiwan	Sawada (1959)
***T.meilingensis***	**Up to 510 × 2.5–4.5**	**7–13**	**Mononematous, dichotomously branched in the conidiophore, septate, echinulate, brown to dark brown**	**Aseptate, globose, verruculose, echinulate, yellow brown to dark brown**	**Freshwater**	**On submerged bamboo culms**	**China, Jiangxi**	**This study**
*T.ramosa*	330‒600 × 8–18	3–5	Mononematous, erect, straight or flexous, septate, dichotomously branched in the above half, dark to reddish brown, verruculose	Dry, catenate, usually in branched, acropetal chains, spherical, dark brown, verruculose, aseptate	Terrestrial	On dead leaves of *Dendrocalamusstrictus*	India, Goa	D’souza et al. (2001)
*T.trechispora*	Up to 1500 × 8–12	5 × 3 (oval) or 4 (spherical)	Erect, olivaceous, septate, everwhere minutely spinulose	Oval or spherical, ornamented with sharp, raised, broken ridges	Terrestrial	On dead wood	Sri Lanka, Peradeniya	Petch (1917)
***T.yunjushanensis***	**Up to 1150 × 3–4**	**7–12**	**Mononematous, dichotomously branched in the conidiophore, septate, echinulate, pale brown to olivaceous**	**Aseptate, spherical, verrucose, echinulate, yellowish brown to dark brown when mature**	**Freshwater**	**On submerged bamboo culms**	**China, Jiangxi**	**This study**

#### ﻿ Trichobotrys yunjushanensis

Taxon classificationFungiPleosporalesDictyosporiaceae

W. J. Zhang & Z. J. Zhai sp. nov.

444485C6-F284-5BC8-A39F-70EC7EBDFD5D

852618

Fig. 3

##### Etymology.

Referring to the collection site of the Yunjushan Mountain in Jiangxi Province, China.

##### Holotype.

HFJAU 10044.

##### Description.

Saprobic on decaying bamboo culms. **Sexual morph**: Undetermined. **Asexual morph**: Hyphomycetous. ***Colonies*** effuse, white, yellow to olivaceous, velvety. ***Mycelium*** mostly superficial, creeping and twining, composed of septate, brown to olivaceous, branched hyphae. ***Conidiophores*** 3‒4 μm wide (x̄ = 3.4 μm, n = 20), up to 1150 μm long, mononematous, erect, straight or flexous, septate, with fertile dichotomously branched, pale brown to olivaceous, verruculose, echinulate, thick-walled. ***Conidiophores branches*** 18–48 × 3–4 μm (x̄ = 29.1 × 3.6 μm, n = 15), sometimes long, fertile, 0‒1(‒2)-septate, verruculose, rough, pale brown. ***Conidiogenous cells*** 6–11 ×3–5 μm (x̄ = 8.5 × 4.0 μm, n = 10), integrated, polyblastic, terminal to subterminal on fertile branches, with several denticulate conidiogenous loci, hyaline to dark brown. ***Conidia*** 7‒12 μm diam (x̄ = 9.3 μm, n = 30), catenate, usually acrogenous or lateral, aseptate, spherical, verrucose, echinulate, sometimes guttulate, yellowish brown to dark brown when mature.

**Figure 3.**
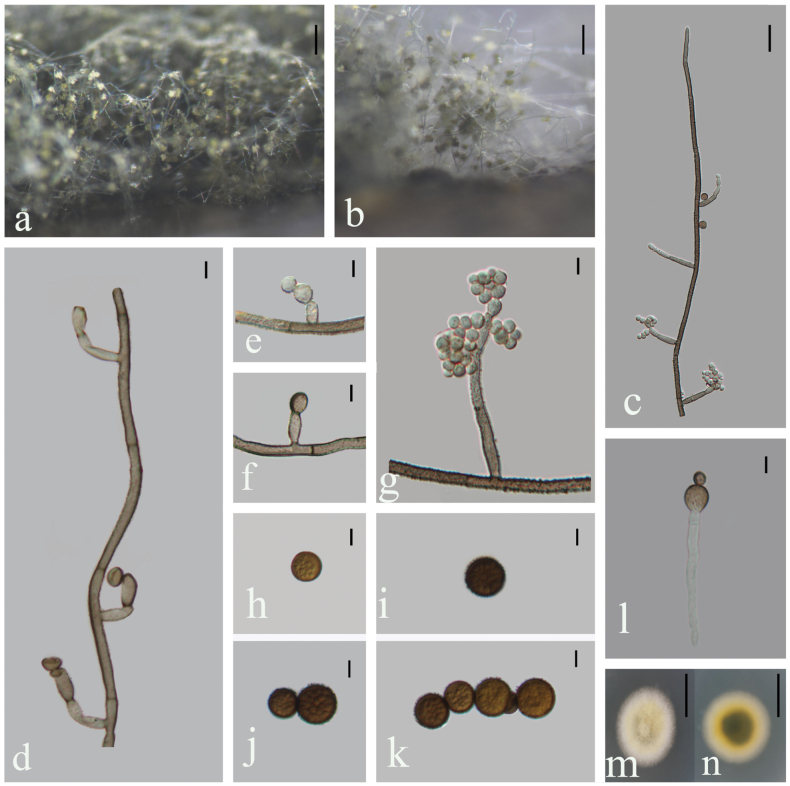
*Trichobotrysyunjushanensis* (HFJAU 10044, holotype) **a, b** colonies on bamboo culm **c, d** conidiophores with conidiogenous cells **e, f** conidiogenous cell with conidia **g** portion of conidiophore with fertile lateral branches **h–k** conidia **l** germinating conidium **m, n** culture on PDA from above (**m**) and reverse (**n**). Scale bars: 100 µm (**a, b**); 20 µm (**c**); 5 µm (**d‒l**); 25 mm (**m, n**).

##### Cultural characteristics.

Conidia germinating on PDA within 24 h. Colonies incubated on PDA media at 25 °C grow rapidly, reaching 21 mm diam after 6 days, in natural light, circular, pale on the margin, yellow at the centre, with white dense aerial mycelium; reverse yellow white to dark green. Hyphae hypline, superficial, septate but not obvious, with a layer of yellow pigment, 1.9‒3.7 μm wide.

##### Material examined.

China. Jiangxi Province: Jiujiang City, Yongxiu County, Yunjushan Mountain, on decaying bamboo culms submerged in a freshwater stream, alt. 672.5 m, 29.23°N, 115.59°E, 28 April 2020, G. P. Xu, Y. Liu and Z. J. Zhai, YJS112 (HFJAU10044, ***holotype***), ex-type living culture, JAUCC 4987 = JAUCC 4988.

##### Notes.

In the multi-gene phylogenetic tree, *Trichobotrysyunjushanensis* groups with *T.effusa* clade with low support (BS/PP = 43/0.67), but they form a monophyletic group when including *T.meilingensis* (Fig. 1). Morphologically, *T.yunjushanensis* is distinct from the holotype of *T.effusa* by its conidial size (7‒12 μm vs. 3‒4 μm) and longer conidiophores (up to 1150 μm vs. up to 200 μm) (Petch 1924). *Trichobotrysyunjushanensis* is mostly similar to *T.meilingensis* and *T.ramosa* in having dichotomously branched and rough conidiophores. However, *T.yunjushanensis* can be easily distinguished from *T.ramosa* by its larger conidia (7‒12 μm vs. 3‒5 μm) (D’Souza and Bhat 2001). Furthermore, *T.yunjushanensis* differs from *T.meilingensis* in having longer conidiophores (up to 1150 μm vs. up to 510 μm) and is phylogenetically distinct from the latter. Therefore, both morphological characters and phylogenetic analyses supported *T.yunjushanensis* as a new taxon within *Trichobotrys*.

#### ﻿ Trichobotrys effusa

Taxon classificationFungiPleosporalesDictyosporiaceae

(Berk. & Br.) Petch, Ann. R. bot. Gdns Peradeniya 9: 169 (1924)

C6620758-2E3B-54E0-82BC-E7E7FB722856

Fig. 4

##### Description.

Saprobic on the stems of decaying wood in freshwater habitat. **Sexual morph**: Undetermined. **Asexual morph**: Hyphomycetous. ***Colonies*** effuse, grayish to nut brown, velvety. ***Mycelium*** mostly superficial, creeping and twining, composed of septate, branched, subhyaline to pale brown hyphae. ***Conidiophores*** 2–4 μm wide (x̄ = 2.7 μm, n = 20), up to 650 μm long, mononematous, erect, straight or somewhat curving, columniform, moderately branched, verruculose, septate, thick-walled, echinulate, light brown to nut brown, gradually attenuated distally to an infertile, setiform apex. ***Conidiophore branches*** 7–26 × 2–4 μm (x̄ = 14.0 × 3.2 μm, n = 16), fertile, 0–1(–2)-septate, verruculose, light brown to dark brown, individual cells typically have a slight swelling. ***Conidiogenous cells*** 3–10.5 × 2.5–6.5 μm (x̄ = 6.6 × 4.0 μm, n = 10), monoblastic or polyblastic, integrated and terminal on lateral branches, apical or lateral; columniform or cannulate, erect or slightly curved, with several seriated conidiogenous locations, light brown to dark brown. ***Conidia*** 3.5–5 μm diam (x̄ = 4.4 μm, n = 30), catenulate, simple or branched apical chains, aseptate, spherical, verruculose, echinulate, sometimes guttulate, transparent to dark brown or red brown.

**Figure 4.**
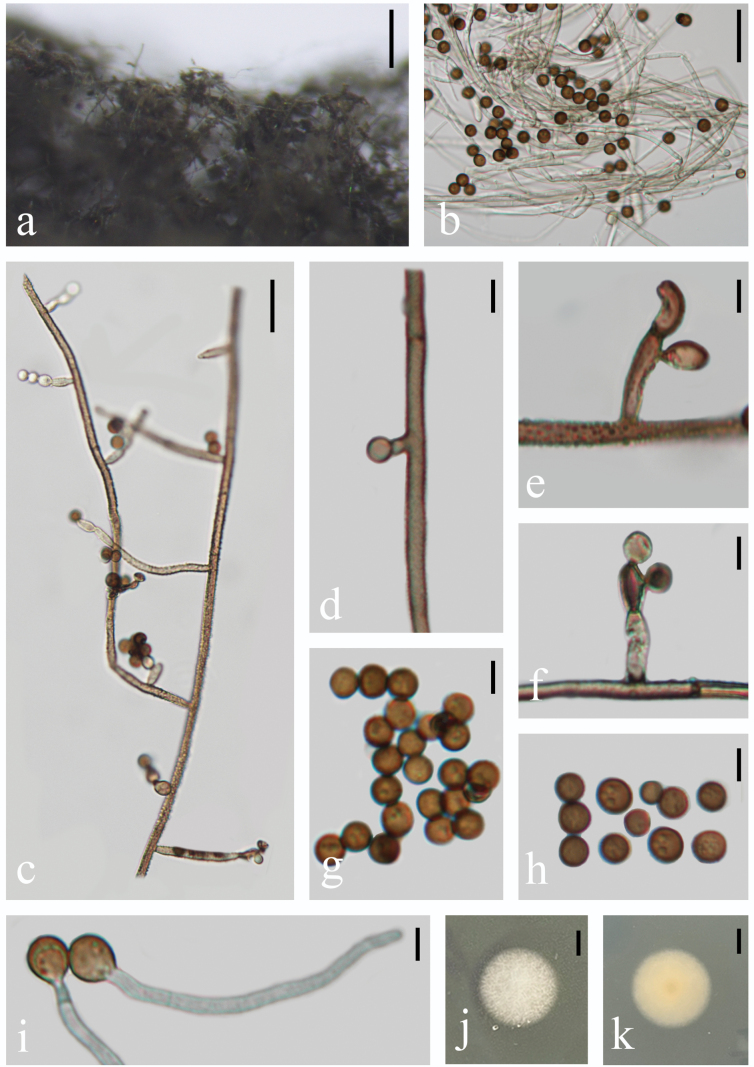
*Trichobotryseffusa* (HFJAU10296, HFJAU10372) **a** colonies on the substrate **b** conidiophores with conidia **c** portion of conidiophore with fertile lateral branches **d–f** conidiogenous cell with conidia **g, h** conidia **i** germinating conidia **j, k** culture on PDA from above (**j**) and reverse (**k**). Scale bars: 100 µm (**a**); 20 µm (**b, c**); 5 µm (**d–i**); 5 mm (**j, k**).

##### Cultural characteristics.

Conidia germinating on PDA within 24 h. Colonies incubated on PDA media at 25 °C attaining 11.5 mm diam after 11 days, in natural light, circular, white, cottony, with white dense aerial mycelium; reverse yellow, white at the margin part.

##### Material examined.

China. Guangxi Province: Guigang City, Pingtianshan National Forest Park, on decaying wood submerged in a freshwater stream, alt. 980.84 m, near 23.19°N, 109.51°E, 11 March 2023 and 16 May 2024, Wan Hu and Z. J. Zhai, HG13 and HG13-1 (HFJAU10296, HFJAU10372), ex-type living culture, JAUCC 6359 = JAUCC 6826.

##### Notes.

According to phylogenetic analysis (Fig. 1), we can find that our new isolates cluster with *Trichobotryseffusa* FS524 and *T.effusa* YMJ1179 with high support (BS/PP = 100/1). Morphologically, our new collections are similar to the holotype of *T.effusa* except for the slightly larger conidia (3.5–5 μm vs. 3–4 μm), longer conidiophores (up to 650 μm vs. up to 200 μm), and slightly different colors in mycelium (grayish to nut-brown vs. dark purple-brown) (Petch 1924). The difference in color might be due to the discrepancy in incubation time and the exposure to light or different observation angles under the microscope. The differences in the size of conidiophores and conidia are also occurring in another record of *T.effusa*, in which the conidiophores and conidia are described as being up to 1000 μm long and 5–7μm in diameter, respectively (Morgan-Jones et al. 1987). The differences among the holotype and our new collections suggest that factors such as habitat and incubation time may influence the size of conidia and conidiophores. Similar observations have also been discovered in the asexual morph of other fungal species (Yang et al. 2018b; Zhang et al. 2022; Shen et al. 2024). Owing to the unavailable molecular sequences in the holotype of *T.effusa* and the deficiency of morphological descriptions about *T.effusa* FS524 and *T.effusa* YMJ1179, the possibility cannot be excluded that our new isolates are a different species to *T.effusa*. However, there are no significant morphological differences between our collections and the holotype. Therefore, we propose to identify the new collections as *T.effusa* until more strains have been examined. The new collection was collected from submerged, decaying wood in Guangxi Province, which is a new discovery in freshwater habitat in China.

## ﻿Discussion

The new isolates *Trichobotryseffusa* (JAUCC 6359 and JAUCC 6826) group well with two strains (FS524 and YMJ1179) of *T.effusa* (BS/PP = 100/1). The high molecular support and morphological similarities among them indicate that they are conspecific and the two isolates (JAUCC 6359 and JAUCC 6826) are identified as a new record of *T.effusa*. Although four-loci data for *T.effusa* FS524 and *T.effusa* YMJ1179 were lacking and they were sequenced only by ITS, our result should be convincing because the fungal ITS marker generally produces considerably more sequence variability, and thus can provide high resolution for species delimitation (Nilsson et al. 2008; Szczepańska et al. 2021). The holotype of *T.effusa* was discovered on dead bamboo from Sri Lanka (Berkeley and Broome 1873; Petch 1924). Subsequently, a series of *T.effusa* strains have been found but were mostly isolated from marine sediment samples collected in the South China Sea (Chen et al. 2014; Sun et al. 2015, 2016; Chen et al. 2020; Liu et al. 2020; Huang et al. 2023), and they were identified as *T.effusa* almost only based on ITS region sequence comparison with the GenBank database. This study is the first report of collection of *T.effusa* from the freshwater habitat and provides both molecular phylogenetical and morphological description for this species.

Two new species, *T.meilingensis* and *T.yunjushanensis*, were proposed as members of *Trichobotrys* based on four-loci (ITS, LSU, SSU and *tef1-α*) phylogenetic analyses in combination with morphological characteristics. However, the relationship between *T.yunjushanensis* and the *T.effusa* clade was unresolved due to low support value. At present, the clade including *T.meilingensis*, *T.yunjushanensis* and *T.effusa* is paraphyletic, therefore, the phylogeny relationships within this clade will become clearer with more new closely related species discovered. Besides, D’Souza and Bhat (2001) described *T.ramosa* from the forest of southern India, but no molecular data of these species are available, so it is difficult to clarify the phylogenetic relationship between this species and other taxa in *Trichobotrys*. However, *T.meilingensis* and *T.yunjushanensis* can be distinguished from *T.ramosa* by morphological characteristics. Detailed information about their morphological comparison can be obtained from the notes and Table 2 in this paper.

*Trichobotrys* appears as sister to *Gregarithecium* with high molecular support and is hence assigned to the family Dictyosporiaceae. The asexual morphs of *Trichobotrys* also mostly resemble other members of Dictyosporiaceae in possessing brown, cheirosporous conidia, produced from holoblastic conidiogenous cells, on micronematous conidiophores (Boonmee et al. 2016). Although we consider that species of *Trichobotrys* are closely related to *Gregarithecium*, the position of *Trichobotrys* in Dictyosporiaceae and relationship between the two genera are still doubtful due to the long branches between *Gregarithecium* and *Trichobotrys* clade and the lack of asexual morph of *Gregarithecium*. Hence, more samples closely related to *Gregarithecium* and *Trichobotrys* are required to be discovered to clarify the position of *Trichobotrys* in Dictyosporiaceae.

It has been widely reported that *Trichobotryseffusa* as the type species of *Trichobotrys* has the ability to produce diverse secondary metabolites (Chen et al. 2014; Chen et al. 2020; Huang et al. 2023; Liu et al. 2020; Sun et al. 2015, 2016). For example, Chen et al. (2014) obtained four novel aliphatic phenolic ethers with growth-inhibitory activity against the A549 lung cancer cell and Sun et al. (2016) received three new macrodiolides with antifouling activity. In this research, we introduce two novel species, *T.meilingensis* and *T.yunjushanensis*, which are both morphologically and phylogenetically similar to *T.effusa*. Furthermore, these two species both can produce yellow pigments and might have the ability to generate secondary metabolites like *T.effusa*. Therefore, future pharmacological evaluation of the two new species might be worth studying to confirm if they are similar to *T.effusa* in having similar bioactive constituents and function in secondary metabolites.

## Supplementary Material

XML Treatment for Trichobotrys meilingensis

XML Treatment for Trichobotrys yunjushanensis

XML Treatment for Trichobotrys effusa

## ﻿Additional information

### Author ORCIDs

Wen-Jing Zhang https://orcid.org/0009-0001-2962-498X

Deng-Mei Fan https://orcid.org/0000-0001-9825-8605

Dian-Ming Hu https://orcid.org/0000-0002-4750-2871

Zhi-Jun Zhai https://orcid.org/0009-0008-7562-9707

### Data availability

All of the data that support the findings of this study are available in the main text.
